# Etiology of Dental Caries

**Published:** 1885-11

**Authors:** Richard White

**Affiliations:** England.


ARTICLE IV.

### ETIOLOGY OF DENTAL CARIES.

BY RICHARD WHITE, L. D. S., ENGLAND.

The histological anatomy of the teeth and their environment has been so thoroughly sifted in the recent past by the aid of the microscope, that Nature can have but few secrets to reveal in this direction. The physiology of the dental tissues has been established on a more or less satisfactory basis, leaving but a limited field of enquiry for future observers, but dental pathology is as yet in its infancy, and offers a magnificent prospect for orginal research. Many and varied are the theories regarding the etiology of caries, greater or less weight being given to the so-called external causes—mechanical, chemical and parasitic, including the action of the food, the buccal secretions and air—while hardly sufficient attention has been bestowed on those more subtle internal agencies, developmental, nervous, vascular and trophic. Moreover, we have heretofore intermingled predisposing and exciting causes. These must be isolated and duly classified before a satisfactory scientific basis can be established. How far parasitic germs can be the *materies morbi* it is not for me to determine, but analogy suggests the probability of their being concomitant with the disease, rather than the true exciting cause of decay. The air is ever impregnated with bacterial germs, and their nourishment being deprived from the products of decomposition, the seats of decay offer a suitable *nidus* for their proliferation.

In the impaired nervous force, the altered qualitative and quantitative blood supply, and other trophic diminutions, we have what seem to be the true predisposing causes of caries. The exact point of origin of the disease may be purely accidental, arising from the agency of any one or more of the so-called external causes, the importance of which must be therefore largely discounted. The elucidation of this complicated subject might be accelerated by experimental research, by ascertaining the results of traction, section and electrical irritation of the inferior dental nerve in animals, experimental alteration of the quanity of blood supplied to the teeth and, when possible, the quality of the same. But perhaps I am anticipating events too rapidly, so let me turn to a more general subject.

No matter connected with our profession is just now attracting more attention than the general deterioration in the structure of the human teeth of the present day. The older members of the profession especially are daily reminded of an alteration in the character of the teeth they are called to operate upon, compared with those of former years —for then usually they found a density of structure which augured well for successful operations, and the results of those operations were most satisfactory—but the teeth of the present period will seldom permit us to promise anything so favourable, from the acknowledge fact that the tissue upon which we have to operate is so defective in the combination of its constituents, although the operative skill employed has of late years immensely improved. To what is this change of structure to be attributed? This opens up a vast field for investigation.

The deterioration in the structure of the teeth which we observe is not confined to one particular class of people in the civilized world. The highly-fed and the poorly-nourished appear to suffer alike. Nor does country or climate apparently exercise much influence upon the animal economy. English. French, Germans and Americans all seem on the decline as regards the durability of these organs. The last named, probably, more than any of the other nations, which from an ordinary view of things, would be considered anomalous—for when we have an admixture of fresh blood, we naturally expect to find a more healthy condition of the progeny—for such physiological characteristics apply to animal nature in every form.

We have reasons to believe that man in a state of nature, free from the strains of mental pressure, and unattacked by the vices that accompany civilization, escapes this morbid condition of the teeth. In the mouths of the wild denizens of the back woods or the dwellers upon lands untouched by civilization, at the present day, or in the exhumed skulls of the inhabitants of this isle in the long distance past, we find well-developed maxillæ, and a bold regular arrangement of thirty-two dense teeth, generally quite free from the ravages of caries; but at the present day we meet with maxillæ extremely contracted and not admitting of the perfect arrangement of the thirty-two teeth when they are erupted. Then deficiencies in the number of teeth are often noticeably for in many cases one or both the upper lateral incisors are absent, and one or more of the upper canines, if found in the jaw, fail to make their appearance through the gum. The wisdom teeth, moreover, are very frequently absent.

This deterioration of the teeth resulting from a departure from the laws of nature, has probably existed among civilized nations for thousands of years; for it is said that the inhabitants of the ancient empires of the world paid the same penalty from a like cause, if not to the same extent as we are now doing. That caries exists in almost every mouth at the present day, and that its victims during the last few years have increased to an alarming extent, we do not doubt, for of that we have daily proofs.

A question naturally arises—what physiological cause has brought about this change of structure? For it appears to be the only portion of the human frame that has exhibited this marked deterioration.

It is not my intention on the present occasion to discuss at length this difficult subject, but to suggest points for investigation that I imagine will amply repay those who have at their disposal the time requisite for such an undertaking.

Those who have had the opportunity of examining many cases of the teeth of persons from four generations of patients (which has fallen to my lot), will, I believe, acknowledge that the teeth in each succeeding generation, as a rule, have become more and more degenerated as regards their structure, and are more prone to be affected by caries, that the teeth of females have deteriorated much more than those of the male sex, and further, that through the female line this deterioration is much more marked than through the male line, for a mother with very defective teeth will invariably have children whose teeth partake of the same character

Acknowledging this deterioration, may not an attempt be made to discover its cause.

That the mind of the mother exercises an immense influence over the constitution of the child when in its fœtal state no one can doubt, and is it not possible that in these days of constant excitement and of general unrest among all classes, that its baneful effects may have an influence upon the rudimentary dental organs of the child at this early stage? And may not also in these days, the high mental pressure permitted, and the artificial life of early childhood continuing in rebellion against nature's laws, carry on the degenerative process that had been so early commenced?

The result of an immoral life handed down from generation to generation tells with fearful effect upon the children of such parents, and the medical treatment in former times to combat the resultant disease, if not at the present day, has had its influence upon the formation of the teeth.

The diet of children during their early years, and until the teeth are fully formed, has been considered of great importance, although in some cases where this matter has been most strictly adhered to, the result has not always, I fear, proved satisfactory. It appears to me, that much cannot be accomplished in this important subject until we are in possession of statistics for our guidance, for without such assistance we are but groping in the dark.

Would it not be possible to ascertain certain facts from our patients which might be tabulated, from which data might be obtained that might guide us, as well as our medical friends, in advising such measures as might, to a certain extent, combat the result of agencies that are at work in producing these deteriorated teeth?

Collective investigation in the hands of the British Medical Association has already largely assisted in unravelling the history of several obscure diseases, and if persevered in, will aid in furthering the rational treatment of disease as opposed to the empirical drugging of the past.

Collective investigation would eminently assist *us* in ascertaining the causes of this general deterioration in the structure of the dental tissues. We might enquire regarding, and record—

(1) The age of patient.
(2) The general health.
(3) The number and general nature of the teeth.
(4) Number diseased.
(5) General health of parents and nature of their teeth.
(6) If consanguinity existed.
(7) Habits and diet of mother at time of birth.
(8) Habits and diet of child in infancy and during juvenescence.

We thus arrive at the subject of prophylactic or preventive dentistry, or, as it may be termed, dental hygiene, that highest scientific branch of our profession in the future, which can alone be established as the outcome of the elucidation of the causes of disease, and will tend to place us higher in the estimation of the general public than the perfecting of any other branch of our art; for they will recognize in the advocacy of its principles that splendid abnegation of self which can have but one aim—the maintenance of the health of the community. We shall then work on the same lines as our medical brethren, who seek to prevent disease as much as to combat it when already existing.—*Dental Record.*

